# XGBPred-ACSM: A Hybrid Descriptor-Driven XGBoost Framework for Anticancer Small Molecule Prediction

**DOI:** 10.3390/ph19040635

**Published:** 2026-04-17

**Authors:** Priya Dharshini Balaji, Subathra Selvam, Anuradha Thiagarajan, Honglae Sohn, Thirumurthy Madhavan

**Affiliations:** 1 Computational Biology Laboratory, Department of Genetic Engineering, School of Bioengineering, SRM Institute of Science and Technology, Kattankulathur, Chengalpattu 603203, Tamil Nadu, India; priyamedigen@gmail.com (P.D.B.); subathraselvam15@gmail.com (S.S.); 2 Department of Physics with Computer Application, Agurchand Manmull Jain College, Meenambakam, Chennai 600061, Tamil Nadu, India; 3 Department of Chemistry, Chosun University, Gwangju 61452, Republic of Korea

**Keywords:** cancer, anticancer small molecules, machine learning, molecular descriptors, fingerprints, XGB

## Abstract

**Background/Objectives**: Cancer remains one of the leading global health burdens, mainly because of the lack of specificity and off-target toxicity associated with conventional therapeutic approaches. To move toward more efficient anticancer drug discovery, we have developed an advanced machine-learning-based architecture that allows for predictive modeling of anticancer small molecules. **Methods**: A total of 3600 compounds with experimentally validated IC_50_ values were systematically processed to derive a comprehensive suite of molecular representations comprising 2D physicochemical descriptors, structural fingerprints, and hybrid descriptor sets generated via the Mordred and PaDEL frameworks. A total of six machine learning algorithms—Random Forest (RF), Extreme Gradient Boosting (XGB), Gradient Boosting (GB), Extra-Trees classifier (ET), Adaptive Boosting (AdaBoost), and Light Gradient Boosting Machine (LightGBM)—were trained and benchmarked via a rigorous model evaluation protocol incorporating 10-fold cross-validation along with multiple performance metrics. Ensemble voting strategies were also examined to assess potential performance. **Result**: Of all configurations, the XGB-Hybrid architecture emerged as the most robust and generalizable classifier with an AUC of 0.88 and accuracy of 79.11% on the independent test set. To ensure interpretability and mechanistic insight, SHAP-based feature analysis was conducted, by which feature contributions could be quantified and the molecular determinants most influential for anticancer activity discrimination were revealed. Altogether, the current study establishes an XGB-Hybrid framework as technically rigorous, interpretable, and high-performance predictive modeling with the ability to accelerate early-stage anticancer small molecule identification. **Conclusions**: The study has brought into focus the transformational effect of machine learning in modern computational oncology and rational drug design pipelines.

## 1. Introduction

Cancer remains one of the most formidable challenges in global healthcare. Despite notable advancements in cancer therapies, the lack of specific drugs for many types of cancer persists as a significant concern [1]. Traditional cancer therapies like radiotherapy and chemotherapy are limited in their ability to directly target cancer cells, often leading to side effects [2]. Consequently, there is an increasing need for the development of more effective and targeted cancer treatments [3]. Small molecule therapeutics plays an essential part in anticancer therapy owing to their relatively favorable pharmacokinetics properties, chemical tractability, and ability to target a wide array of intracellular targets involved in tumor formation and growth. Compared to biologics, small molecules have better oral bioavailability, tissue penetrability, and target accessibility, making them a suitable option for targeted and combination anticancer therapies [4,5]. However, the identification of anticancer small molecules remains challenging and faces many challenges, since screening large libraries by biological assays is a labor-intensive and time-consuming process, making it prohibitively expensive [6,7]. To address these challenges, we designed a robust computationally intelligent framework leveraging machine learning (ML) for predicting anticancer small molecules (ACSMs) [8,9,10,11]. ML models utilize high-dimensional datasets, involving molecular descriptor vectors originated from chemical structure and experimentally derived bioactivity data, to derive complex structure–activity relationships that are too often difficult to define with the application of heuristic or rule-based methodology [12,13,14]. Although ML does not eliminate the need for experimental validation, it provides useful computational guidance through compound library prioritization, biological activity prediction, and selection of the most promising compound candidates for further investigation. Trained against curated bioactivity datasets, ML-based strategies have shown great potential to significantly reduce experimental burdens by focusing limited resources on high-confidence predictions, saving time, cost, and effort at multiple stages of the anticancer drug discovery process [15]. Anticancer small molecules (ACSMs) offer significant structural diversity and physicochemical variability, aiding exploration of diverse mechanisms and molecular targets. Such diversity makes ACSMs particularly suitable for descriptor-based and ensemble machine learning frameworks aimed at predictive modeling. Given the continued need for efficient computational screening strategies in anticancer research, we leveraged machine learning methodologies to construct predictive models for anticancer small molecule identification [16].

In the previous MLASM study, we developed baseline ML models using five conventional algorithms that were trained only on 2D molecular descriptors. While those models showed promising results, their predictive ability was limited by the restricted feature representation. The present work offers a much-improved and extended version of the previous framework [9].

The goal of our present study is to develop high-performance classifiers for anticancer small molecules based on enhanced feature representation and extended algorithmic strategy. For this purpose, six machine learning algorithms, namely, Random Forest (RF), Extreme Gradient Boosting (XGB), Gradient Boosting (GB), Extra-Trees classifier (ET), Adaptive Boosting (AdaBoost), and Light Gradient Boosting Machine (LightGBM), were employed together with ensemble learning using soft and hard voting. We explored three different feature combinations: (i) 2D molecular descriptors, (ii) structural fingerprints, and (iii) hybrid features combining both types of descriptors. To enhance the discriminatory abilities of these models, we used SVC-L1 feature selection to retain the most informative molecular attributes. Further, SHapley Additive exPlanations (SHAP) analysis was carried out to identify key descriptors responsible for model decisions with a view to strengthening model interpretability. Overall, these methodological enhancements—broader feature engineering, expanded algorithmic diversity, incorporation of ensemble learning, and explainable AI analysis—represent a considerable evolution compared to our prior work and considerably enhance the predictive modeling of anticancer small molecules [17].

## 2. Results and Discussion

### 2.1. Dataset Analysis and Feature Selection

**Figure 1:**
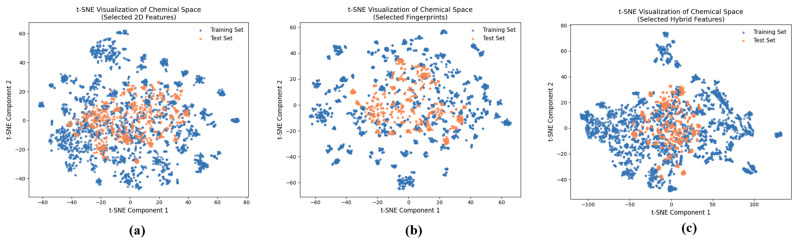
t-SNE Visualization of Chemical Space: (**a**) Selected 2D Features, (**b**) Selected Fingerprints, and (**c**) Selected Hybrid Features.

**Figure 2:**
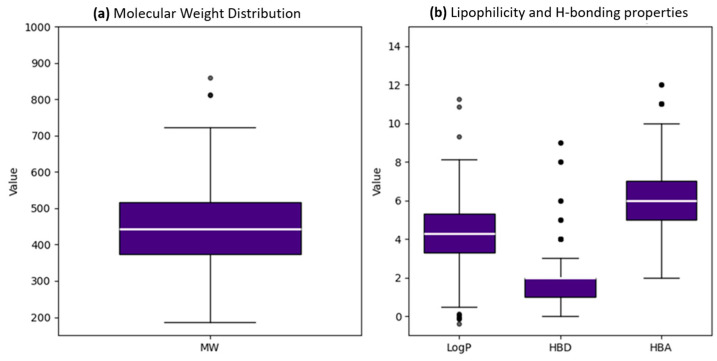
Physicochemical property distributions of the anticancer small molecule (ACSM) dataset. (**a**) Molecular weight (MW) distribution. (**b**) Distributions of lipophilicity (LogP), hydrogen bond donors (HBD), and hydrogen bond acceptors (HBA).

The analysis of structural diversity and chemical space within the dataset is crucial for understanding the predictive capacity of ML models. In this study, we conducted a comprehensive analysis of dataset diversity using t-distributed stochastic neighbor embedding (t-SNE), as depicted in Figure 1. The visualization in Figure 1 illustrates the wide chemical space encompassed by the dataset, encompassing a range of molecular structures and properties. Notably, the training and test sets, represented by the selected 2D features, fingerprints, and hybrid features, share a similar chemical space, indicating consistency and representativeness across the datasets. We can observe that molecules in the training set predominantly occupy the outer regions of the chemical space, while the curated test set molecules are centrally located. This spatial distribution suggests that the training set encompasses a diverse range of molecular structures, potentially capturing a broader spectrum of chemical characteristics. Conversely, the central positioning of the test set molecules signifies a balanced representation within the chemical space, indicating reliability and generalizability in model performance evaluation. To further analyze the chemical space of the anticancer small molecule dataset, the key physicochemical properties of the compounds were characterized. Figure 2 illustrates the distribution of molecular weight (MW), lipophilicity (LogP), hydrogen bond donors (HBDs) and hydrogen bond acceptors (HBAs). The distribution of MW (Figure 2a) suggests that most of the compounds fall within a drug-like molecular weight range, with a limited number of higher-molecular-weight outliers. The distribution of LogP, HBD, and HBA (Figure 2b) exhibited a moderate lipophilicity with a balanced hydrogen-bonding capacity (HB), suggesting a good profile for cellular permeability and interaction with a target. Overall, these physicochemical profiles confirm that the dataset covers a chemically diverse yet drug-relevant space suitable for machine-learning-based anticancer activity prediction [18].

**Figure 3:**
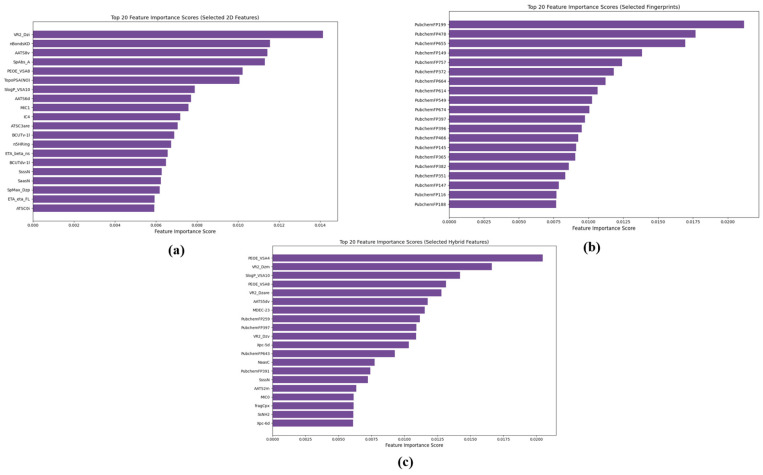
Top 20 Feature Importance Scores: (**a**) Selected 2D Features, (**b**) Selected Fingerprints, and (**c**) Selected Hybrid Features, showcasing the most influential features in the model.

For feature selection, SVC-L1 was used to identify crucial features for model development. This methodology resulted in the identification of 726 2D features and 369 FP features and 1167 hybrid features. Subsequently, we employed six ML algorithms for model construction. To enhance model performance, we incorporated ensemble learning techniques, leveraging XGB and GB for voting. Further, to gain insights into feature importance, we utilized XGB to calculate feature importance scores. The top 20 features from the selected 2D features, fingerprints, and hybrid features were identified and are separately illustrated in Figure 3. This visualization showcases the most influential features in our models, providing a clear understanding of the factors driving predictive performance and model interpretability.

### 2.2. Performance of Classification Models

#### 2.2.1. Analysis of 2D/FP Descriptors

Several ML models were developed to predict ACSM using various chemical descriptors, including 2D and FP descriptors. Multiple ML approaches were employed, including RF, XGB, GB, ET, AdaBoost, and LightGBM, with ensemble learning using XGB and GB for voting. These models were optimized by tuning different parameters on the training dataset using 10-fold cross-validation and evaluated using the test set. Initially, machine learning models were developed using 726 significant 2D descriptors identified through the SVC-L1 feature selection method. Among these classifiers, the XGB model achieved an AUC of 0.89 and an accuracy of 79.90% on the training set and an AUC of 0.85 and an accuracy of 75.42% on the test set. Similarly, the models developed using LightGBM and the voting classifier also demonstrated comparable accuracy. However, when comparing sensitivity, specificity, and MCC, the XGB model outperformed the others, exhibiting balanced sensitivity, specificity, and MCC values (Table 1). Additionally, models were also developed using 369 FP descriptors selected from SVC-L1, and from these we have found a slight difference in the GB-based model, which outperformed other methods by achieving an AUC of 0.89 and an accuracy of 79.83% on the training set, as well as an AUC of 0.86 and an accuracy of 73.89% on the test set (Table 2). Though the XGB model exhibited strong individual performance in both 2D and FP descriptors, GB performed very similarly according to several metrics for the fingerprint-based models. The general performance of both models was stable for the training and independent test set predictions, with relatively well-balanced sensitivity and specificity. Therefore, a soft-voting ensemble was used to combine these two robust predictors (XGB and GB) to enhance robustness and reduce variance in prediction.

#### 2.2.2. Analysis of Hybrid Features

**Figure 4:**
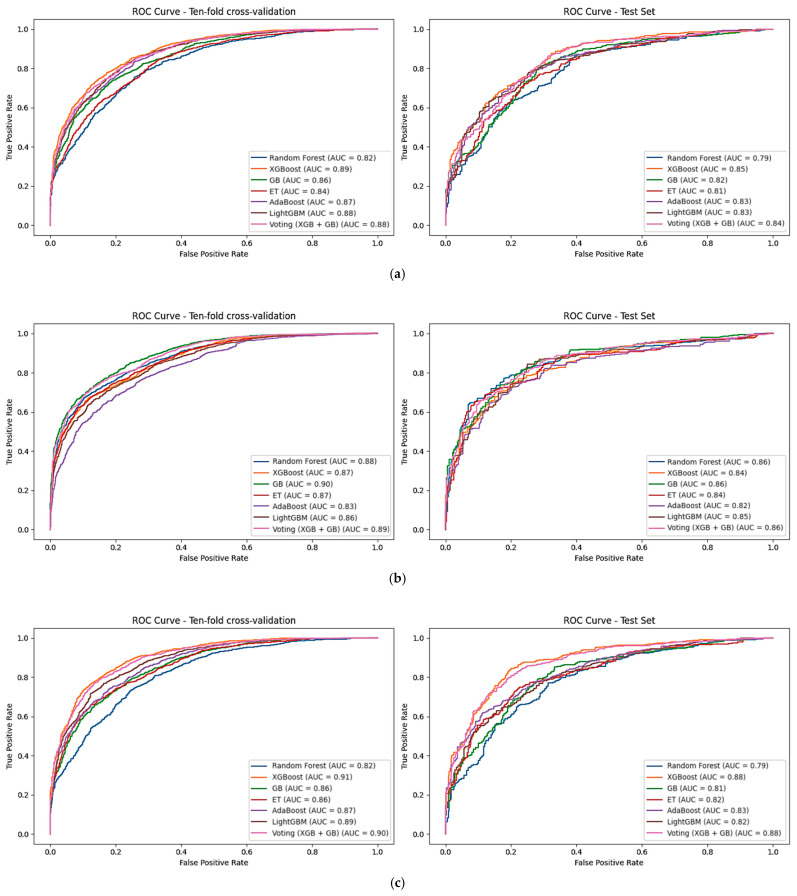
ROC Curve Comparison of Five Machine Learning Algorithms on Anticancer Small Molecules: Demonstrating Receiver Operating Characteristic (ROC) curves from 10-fold cross-validation and Test set. The algorithms include RF, XGB, GB, ET, AdaBoost, LightGBM, and Voting (XGB + GB), with (**a**) Selected 2D Features, (**b**) Selected Fingerprints, and (**c**) Selected Hybrid Features.

**Figure 5:**
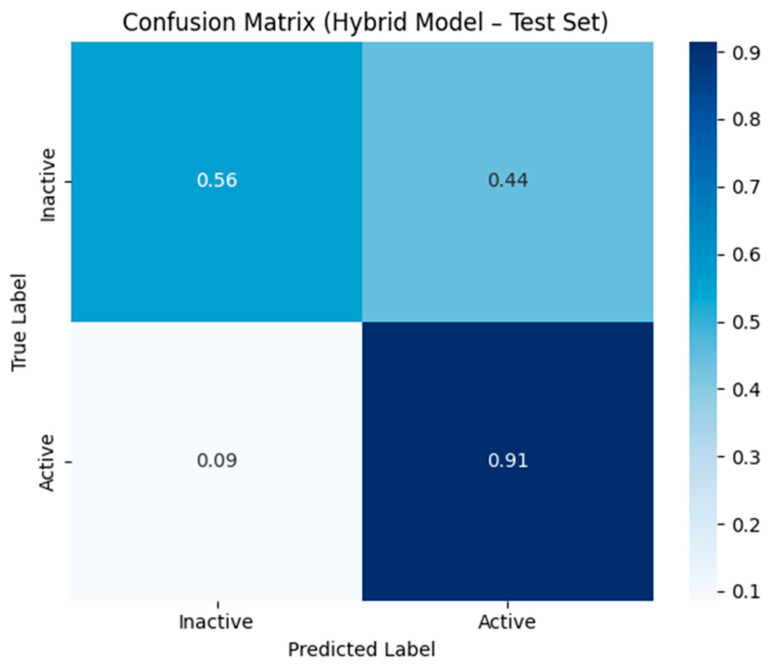
Confusion matrix of the hybrid XGB model evaluated on the test set.

In addition to developing prediction models using single descriptors, we also constructed an ML-based hybrid model by integrating both types of descriptors, namely 2D and FP. The hybrid models were developed using a total of 1167 features. Consistent with observation from the individual descriptor analyses, the hybrid feature results improve the applicability of the ensemble approach utilized (XGB-GB) in the prediction of robust and reliable anticancer compounds. Notably, the XGB-based hybrid model achieved an impressive AUC of 0.90 and an accuracy of 82.05% on the training set, as well as an AUC of 0.88 and an accuracy of 79.11% on the test set, demonstrating balanced sensitivity and specificity in identifying ACSM (Table 3). The results indicate that the inclusion of hybrid features contributes significantly to achieving optimal performance, making it the top-performing model. Moreover, we conducted a comprehensive analysis of the ROC curves for each classifier, illustrating their performance across various thresholds. The XGB model exhibited superior performance in terms of ROC curve characteristics, highlighting its effectiveness in distinguishing between active and inactive anticancer compounds using the selected 2D, FP, and hybrid features (Figure 4). For the final hybrid model, additional screening-relevant performance metrics were calculated on the test set using a fixed probability threshold of 0.5. The model exhibited a precision of 0.67, recall of 0.92 and an F1-score of 0.77, reflecting its high capability in identifying active compounds Similarly, PR-AUC confirmed the appropriateness of the model for virtual screening applications (Table 4). Confidence intervals for AUC and MCC were estimated by applying bootstrap resampling with 1000 iterations to measure the robustness of predictive performance. The corresponding confusion matrix illustrating the classification outcome for the hybrid model test set is shown in Figure 5. Additionally, we performed feature importance analysis on the hybrid model, identifying key descriptors that significantly contributed to its predictive power. This analysis provided valuable insights into the underlying chemical properties driving the model’s performance and further validated the effectiveness of the hybrid approach in capturing relevant information for anticancer compound prediction.

Although descriptor free approaches, such as graph neural networks and SMILES-based deep learning have exhibited good predictive performance, the present study mainly focused on descriptor-based machine learning techniques. These models also provide enhanced interpretability, lower computational time, and improved reproducibility, specifically for datasets with moderate numbers. In addition, descriptors allow the direct analysis of structure–activity relationships and feature importance interpretation, which is in accordance with the objective of anticancer small molecules screening [19].

#### 2.2.3. Performance Under Scaffold-Based Validation

Bemis–Murcko scaffold-based validation was conducted to further evaluate the generalizability of the developed model across structurally diverse compounds. In this analysis, compounds were grouped according to their core Bemis–Murcko scaffolds to ensure that molecules sharing the same scaffold were not simultaneously present in both the training and test sets. This technique provides a more rigorous evaluation of model performance by assessing its ability to predict bioactivity for previously unseen chemical frameworks. The scaffold-based evaluation was conducted for the best-performing hybrid XGB model, which achieved an AUC of 0.84 and an accuracy of 76.40%, with balanced sensitivity (78.33%) and specificity (74.43%). The model also demonstrated stable predictive capability with an MCC of 0.53, indicating reliable classification performance even when evaluated on structurally novel compounds. These findings suggest that the proposed framework maintains robust predictive capability beyond closely related molecular structures. The detailed performance metrics obtained from the scaffold-based evaluation are provided in Table S2 (Supplementary Materials).

### 2.3. Feature Analysis

**Figure 6:**
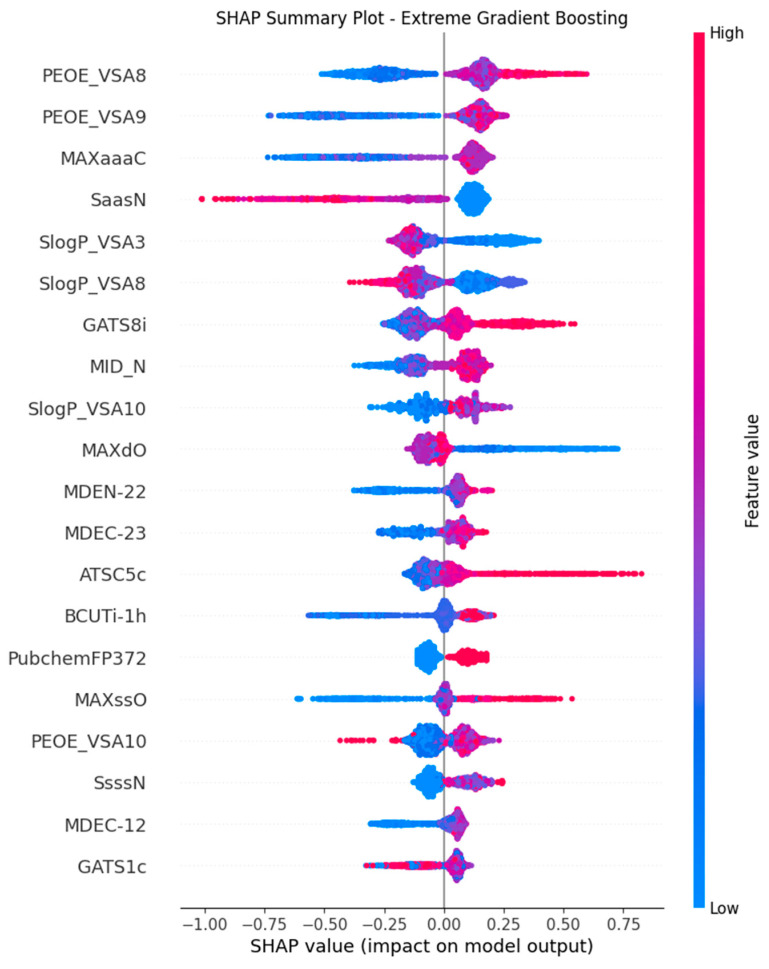
SHAP Summary Plot for Feature Importance in the Extreme Gradient Boosting (XGB) Model. The bar plot illustrates the impact of individual features on model predictions.

In our study, we conducted a comprehensive feature analysis using SHAP plots to interpret the top-performing XGB-based hybrid model, integrating 2D and FP descriptors. The SHAP analysis, applied to our XGB, revealed the significance of each feature in the model predictions (Figure 6). Each dot in the SHAP summary plot represents a Shapley value, indicating the feature’s impact on the model’s predictions. The color of the dots represents feature values, with red indicating high values and blue indicating low values, while their position along the y-axis reflects the magnitude and direction of the contribution [17]. The identified hybrid features provided detailed insights into the molecular characteristics driving the anticancer potential of small molecules. The electrostatic surface descriptors (PEOE_VSA8, PEOE_VSA9) were identified as a most influential feature. These surface descriptors relate to partial charge distribution over certain surface areas of the molecule and are associated with molecular interaction properties. Hydrophobic surface descriptors (SlogP_VSA3, SlogP_VSA8) proposed that these surface descriptors relate to the lipophilicity of small molecules, thereby possibly influencing their permeability and accessibility within the cell. Structural and topological descriptors (MDEC-12, MDEC-23) describe information associated with patterns of molecular distances, which may be relevant to conformational flexibility. Other graph-theoretical descriptors such as MAXaaaC, GATS8i, and GATS1c describe atomic connectivity and property distribution patterns, which are often present in bioactive chemical compounds. Further, descriptors associated with substructural features and drug-likeness, such as BCUTc-1h, MAXssO, Pub-chemFP372, and XlogP, describe features commonly associated with bioactive compounds in cancer libraries. Overall, the SHAP-based analysis therefore provides interpretable insights into the molecular characteristics influencing model predictions.

## 3. Materials and Methods

### 3.1. Dataset Construction

A total of 3600 anticancer small molecules, along with their IC_50_ values, were collected from the PubChem BioAssay database. We specifically focused on assays designed to evaluate anticancer activity, encompassing cell-based proliferation and cytotoxicity assays as well as biochemical assays that measure target-specific inhibition, particularly against a well-established oncogenic target, including protein kinases and key enzymes involved in DNA repair pathways [20]. To maintain biological relevance and transparency, specifically human-derived assays reporting quantitative IC_50_ values were used. The curated dataset includes both cell-based antiproliferative/cytotoxicity assays (e.g., HT-29, MDA-MB-231, A-375, HCT116, MCF-7, K562, A2780) and biochemical assays targeting cancer-related proteins. A representative list of PubChem BioAssay identifiers (AIDs), assay types, biological targets or cell lines, and readout formats is given in Table S1 (Supplementary Materials). A schematic outline of the dataset curation workflow, including assay filtering, duplicate removal, IC_50_ transformation, and class labeling, is illustrated in Figure S1 (Supplementary Materials). When multiple IC_50_ values were available for a compound within comparable assay contexts, a single representative IC_50_ value was retained to avoid redundancy. No aggregation across mechanistically unrelated targets was performed. This approach minimizes potential label noise arising from heterogeneous assay contexts and improves biological consistency of the classification task. Subsequently, IC_50_ values were transformed into their respective pIC_50_ values, using the standard formula of pIC_50_ = −log10(IC_50_). This transformation is widely used for the normalization of activity values, particularly in QSAR and machine learning models, to ensure improved numerical stability [21]. Following the transformation, the pIC_50_ values ranged from 3 to 10, indicating the compounds have varying anticancer potencies from weak to high activity. Compounds with pIC_50_ >= 7 (equivalent to IC_50_ ≤ 100 nM) were classified as active, while those with pIC_50_ < 7 were labeled as inactive. This threshold is widely implemented in anticancer virtual screening and QSAR-based classification studies to differentiate highly potent compounds suitable for lead identification from moderately or weakly active molecules. Application of this cutoff resulted in 1800 active and 1800 inactive compounds, thus providing balanced class distribution. Since no considerable class imbalance was observed, no further resampling or class weighting strategy and threshold adjustment techniques were necessary during model training [22]. Further, the dataset was initially divided using a random 80:20 split, where 80% of the data were used for training, while the remaining 20% were used for validation and additional scaffold-based validation was performed to assess model generalization across structurally diverse chemical scaffolds [23,24].

### 3.2. Feature Calculation

Choosing suitable molecular representations is indeed crucial for developing robust ML models. It ensures that the models can effectively identify and learn relevant features and patterns within the data, resulting in more accurate predictions and reliable outcomes [25]. In this study two key tools, namely PaDEL (v2.21) [26] and Mordred (v1.2.0) [27], were employed to compute molecular fingerprints and 2D molecular descriptors for 3600 compounds based on their simplified molecular-input line-entry system (SMILES) representation. Specifically, an 881-bit binary PubChem fingerprint (FP) was computed using PaDEL-descriptor, while 1613 2D molecular descriptors were generated using Mordred.

Prior to model development, descriptor preprocessing was employed on the training dataset to prevent information leaks. Continuous molecular descriptor z-score standardization was applied, in which zero mean and unit variance are used, where the parameters are fit exclusively based on the training data, then applied to the independent test set. Descriptors with over 60% missing values were removed, while the remaining descriptors had their missing values replaced using the mean imputation method, which is suitable for continuous descriptor data and conserves overall feature distribution, without the occurrence of bias. These descriptors were crucial in developing diverse ML models [28].

### 3.3. Feature Selection

Previous research has demonstrated that not all features are equally important, presenting a significant challenge in selecting the relevant features from a large feature set [29]. In this study, we utilized the Support Vector Classifier (SVC) with L1-based feature selection from the scikit-learn library. This method utilizes SVC with a linear kernel and applies L1 regularization to penalize less informative features, thereby effectively selecting the most important features from the high-dimensional feature set. Due to this sparsity-enforcing mechanism, highly correlated and zero-variance descriptors are automatically penalized and removed. The result consists of reduced redundancy, improved numerical stability, and enhanced generalization of the machine learning models.

Applying this SVC-L1 feature selection to our dataset, a total of 726 informative 2D descriptors and 369 FP features were retained. Furthermore, we integrated 2D and FP descriptors to create a hybrid model through SVC-L1 feature selection, and we identified a set of 1167 essential descriptors encompassing both 2D and FP features for model development. The SVC-L1 feature selection was performed specifically on the training data to prevent information leakage. The independent test set was held out and used for model optimization [26,30].

### 3.4. Machine Learning Algorithms and Model Construction

Six ML algorithms were employed to develop classification models, including RF [31], XGB [32], GB [33], ET [34], AdaBoost [35], and LightGBM [36]. Additionally, for ensemble learning, voting was used to combine the predictions of the two best-performing ML models. Furthermore, hyperparameter optimization was employed with the objective of maximizing prediction accuracy [37].

### 3.5. Cross-Validation and Performance Metrics

In this study, the datasets were divided into an 80:20 ratio, where 80% constituted the training set and 20% were allocated for validation. We applied a 10-fold cross-validation (CV) technique to the training data to assess the ML models. Internally, we split the training data into ten folds, using nine for training and one for testing. This process was repeated ten times, ensuring each fold was tested at least once [38]. Hyperparameter tuning and model selection were carried out using 10-fold cross-validation on the training set. The best-performing configuration based on mean cross-validation AUC was then tested on the independent test set.

The performance of various ML models was assessed using standard evaluation metrics, including threshold-dependent and independent parameters [39]. The area under the receiver operating characteristic curve (AUROC) served as a threshold-independent parameter, while sensitivity (SN), specificity (SP), accuracy (ACC), and Matthew’s correlation coefficient (MCC) were threshold-dependent parameters. For all models, a fixed probability threshold of 0.5 was applied to convert predicted probabilities into class labels during binary classification. These metrics have been extensively discussed and annotated in previous studies.

$$\begin{array}{c} \begin{array}{cc} & \;\mathrm{SN}\;=\frac{TP}{TP+FN}\times 100\\ & \mathrm{SP}\;=\frac{TN}{TN+FP}\times 100\\ & ACC=\frac{TP+TN}{TP+TN+FP+FN}\times 100\\ & MCC=\frac{TP\times TN-FP\times FN}{\sqrt{\left(TP+FN)(TP+FP)(TN+FN)(TN+FP\right)}} \end{array} \end{array}$$

where TP represents correct positive predictions, TN represents correct negative predictions, FP represents false positive predictions (negative), and FN represents false negative predictions (positive) [40,41].

### 3.6. Scaffold-Based Validation

To further assess the generalizability of the developed ML models for structures outside of closely related chemical structures, an additional scaffold-based validation was carried out using the Bemis–Murcko framework. Bemis–Murcko scaffolds were generated for each compound in the dataset using its respective SMILES notation and through utilization of the RDKit cheminformatics toolkit. Compounds were grouped into clusters where all compounds were represented by the same scaffold and were then assigned entirely to either the training set or test set to avoid any overlap between the two groups. Following scaffold generation, the dataset was partitioned using an 80:20 scaffold-based split, where approximately 80% of the unique scaffolds were used for model training and the remaining 20% were used for testing. Subsequently, the same pre-processing techniques and feature selection method (SVC-L1) were applied to the scaffold-based ML model generation. This protocol permitted a direct comparison between the conventional random split evaluation and the more rigorous scaffold-based validation strategy [42].

## 4. Conclusions

The incorporation of ML-based approaches into anticancer drug discovery is a powerful strategy to overcome limitations intrinsic in conventional strategies, leading to new cancer treatments. In this research we aimed at building predictive ML models using anticancer small molecule datasets, utilizing three molecular representations, molecular descriptors, fingerprints, and hybrid features, with six well-established ML algorithms involving ensemble learning. Of these models, the XGB-Hybrid model had the best performance, especially in anticancer small molecules’ bioactivity prediction, outperforming models using only molecular descriptors or fingerprints. Incorporating SHAP analysis gave deeper insights into importance features, validating the power of machine learning in bioactivity prediction in the anticancer field. These results contribute to the area of predictive modeling for anticancer small molecules, illuminating the effectiveness of machine learning strategies in drug discovery. The focus on interpretability, as exemplified by SHAP analysis, maximizes model transparency, and allows for informed decision making towards the discovery of new anticancer small molecule inhibitors. We hope that this research will benefit the broader anticancer research community as a means of predicting and validating anticancer small molecules for drug discovery and development.

## Figures and Tables

**Table 1:** Performance Metrics Comparison of ML Algorithms on Anticancer Small Molecules on 10-fold cross-validation and test set with 726 best 2D features: Evaluating Accuracy, AUC, Sensitivity, Specificity and MCC.

Classifier	10-Fold Cross-Validation					Test Set				
	Accuracy (%)	Sensitivity (%)	Specificity (%)	AUC	MCC	Accuracy (%)	Sensitivity (%)	Specificity (%)	AUC	MCC
RF	74.72	76.88	72.57	0.82	0.49	72.50	82.78	62.22	0.79	0.45
XGB	79.90	82.92	76.88	0.89	0.59	75.42	92.78	58.06	0.85	0.54
GB	76.60	79.58	73.61	0.86	0.53	74.31	88.89	59.72	0.81	0.50
ET	74.97	78.82	71.11	0.83	0.50	72.36	87.78	56.94	0.80	0.47
AdaBoost	78.09	80.14	76.04	0.87	0.56	70.97	89.17	52.27	0.83	0.45
LightGBM	79.17	80.90	77.43	0.87	0.58	71.67	87.78	55.56	0.82	0.45
Voting (XGB + GB)	79.24	82.36	76.11	0.88	0.58	75.42	91.67	59.17	0.84	0.53

(Random Forest (RF), Extreme Gradient Boosting (XGB), Gradient Boosting (GB), Extra Trees (ET), Adaptive Boosting (AdaBoost), Light gradient boosting machine (LightGBM), Matthew’s correlation coefficient (MCC), Area under the receiver operating characteristic curve (AUC)).

**Table 2:** Performance Metrics Comparison of ML Algorithms on Anticancer Small Molecules on 10-fold cross-validation and test set with 369 best FPs: Evaluating Accuracy, AUC, Sensitivity, Specificity and MCC.

Classifier	10-Fold Cross-Validation					Test Set				
	Accuracy (%)	Sensitivity (%)	Specificity (%)	AUC	MCC	Accuracy (%)	Sensitivity (%)	Specificity (%)	AUC	MCC
RF	78.30	77.85	78.75	0.87	0.56	72.64	90.00	55.28	0.85	0.48
XGB	75.94	78.12	73.75	0.86	0.51	72.22	87.78	56.67	0.83	0.46
GB	79.83	80.28	79.37	0.89	0.59	73.89	91.94	55.83	0.86	0.51
ET	77.08	76.60	77.57	0.86	0.54	75.00	89.17	60.83	0.84	0.52
AdaBoost	74.20	76.18	72.22	0.82	0.48	72.64	86.94	58.33	0.82	0.47
LightGBM	75.94	76.81	75.07	0.85	0.51	74.44	89.44	59.44	0.84	0.51
Voting (GB + XGB)	78.78	80.00	77.57	0.88	0.57	73.06	90.28	55.83	0.85	0.49

(Random Forest (RF), Extreme Gradient Boosting (XGB), Gradient Boosting (GB), Extra Trees (ET), Adaptive Boosting (AdaBoost), Light gradient boosting machine (LightGBM), Fingerprints (FPs), Matthew’s correlation coefficient (MCC), Area under the receiver operating characteristic curve (AUC)).

**Table 3:** Performance Metrics Comparison of ML Algorithms on Anticancer Small Molecules on 10-fold cross-validation and test set with 1167 best Hybrid features: Evaluating Accuracy, AUC, Sensitivity, Specificity and MCC.

Classifier	10-Fold Cross-Validation					Test Set				
	Accuracy (%)	Sensitivity (%)	Specificity (%)	AUC	MCC	Accuracy (%)	Sensitivity (%)	Specificity (%)	AUC	MCC
RF	74.20	75.56	72.85	0.82	0.48	71.11	81.11	61.11	0.78	0.43
XGB	82.05	83.82	80.28	0.90	0.64	79.11	92.78	59.44	0.88	0.55
GB	76.77	80.07	73.47	0.85	0.53	73.75	87.78	59.72	0.81	0.49
ET	75.69	79.37	72.01	0.85	0.51	69.44	85.00	53.89	0.81	0.40
AdaBoost	77.57	80.42	74.72	0.87	0.55	71.53	88.33	54.72	0.83	0.45
LightGBM	79.55	81.81	77.29	0.88	0.59	69.86	87.78	51.94	0.81	0.42
Voting (GB + XGB)	81.18	83.54	78.82	0.90	0.62	75.69	92.22	59.17	0.87	0.54

(Random Forest (RF), Extreme Gradient Boosting (XGB), Gradient Boosting (GB), Extra Trees (ET), Adaptive Boosting (AdaBoost), Light gradient boosting machine (LightGBM), Matthew’s correlation coefficient (MCC), Area under the receiver operating characteristic curve (AUC)).

**Table 4:** Extended performance evaluation of the hybrid XGB model on test set: Evaluating Precision, recall, F1-score, PR-AUC, AUC (95% CI), and MCC (95% CI).

XGB Model	
Metric	Value
**Precision**	0.67
**Recall**	0.92
**F1-score**	0.77
**PR-AUC**	0.88
**AUC (95% CI)**	0.88 [0.85, 0.90]
**MCC (95% CI)**	0.55 [0.49, 0.61]

(Extreme Gradient Boosting (XGB), Matthew’s correlation coefficient (MCC), Area under the receiver operating characteristic curve (AUC)).

## Data Availability

The original contributions presented in this study are included in the article/Supplementary Materials. Further inquiries can be directed to the corresponding authors.

## Supplementary Materials

The following supporting information can be downloaded at: https://www.mdpi.com/article/10.3390/ph19040635/s1.
