# Supplementary material for: XGBPred-ACSM: A Hybrid Descriptor-Driven XGBoost Framework for Anticancer Small Molecule Prediction
Source: Pharmaceuticals (Basel). 2026 Apr 17;19(4):635. doi: 10.3390/ph19040635 (PMC13118636; doi:10.3390/ph19040635)

Table S1. Summary of curated PubChem BioAssays, including assay type, target or cell line, and IC_{50} readout used for model development.

PubChem AID	Assay Type	Target / Assay Description	Species	Readout
1676058	Cell-based phenotypic assay	Antiproliferative activity measured by cell viability assay in HT-29 colorectal carcinoma cells	Homo sapiens	IC_{50} (nM)
1767176	Cell-based phenotypic assay	Antiproliferative activity in MDA-MB-231 breast carcinoma cells	Homo sapiens	IC_{50} (nM)
1737899	Cell-based phenotypic assay	Antiproliferative activity in A-375 melanoma cells	Homo sapiens	IC_{50} (nM)
452430	Cell-based phenotypic assay	Antiproliferative activity in K562 chronic myelogenous leukemia cells	Homo sapiens	IC_{50} (nM)
1741862	Cell-based phenotypic assay	Antiproliferative activity in MDA-MB-231 breast carcinoma cells	Homo sapiens	IC_{50} (nM)
1755706	Cell-based phenotypic assay	Antiproliferative activity measured by MTT viability assay in PANC-1 pancreatic carcinoma cells	Homo sapiens	IC_{50} (nM)
1471416	Cell-based phenotypic assay	Antiproliferative activity measured by MTT viability assay in HL-60 promyelocytic leukemia cells	Homo sapiens	IC_{50} (nM)
1765385	Cell-based phenotypic assay	Antiproliferative activity in MCF-7 breast carcinoma cells	Homo sapiens	IC_{50} (nM)
1338711	Cell-based phenotypic assay	Antiproliferative activity in HCT116 colorectal carcinoma cells	Homo sapiens	IC_{50} (nM)
1706707	Cell-based phenotypic assay	Antiproliferative activity in K562 chronic myelogenous leukemia cells	Homo sapiens	IC_{50} (nM)
1505717	Cell-based phenotypic assay	Antiproliferative activity in MDA-MB-231 breast carcinoma cells	Homo sapiens	IC_{50} (nM)
321758	Cell-based phenotypic assay	Antiproliferative activity measured by MTT viability assay in HCT116 colorectal carcinoma cells	Homo sapiens	IC_{50} (nM)
275908	Cell-based phenotypic assay	Antiproliferative activity measured by MTT viability assay in MM96L melanoma cells	Homo sapiens	IC_{50} (nM)
483584	Cell-based phenotypic assay	Antiproliferative activity in A2780 ovarian carcinoma cells	Homo sapiens	IC_{50} (nM)
1750230	Cell-based phenotypic assay	Antiproliferative activity measured by MTT viability assay in HeLa cervical carcinoma cells	Homo sapiens	IC_{50} (nM)
53350	Biochemical inhibition assay	Inhibition of Cyclin-dependent kinase 1 (CDK1)–Cyclin B complex	Homo sapiens	IC_{50} (nM)
69423	Biochemical inhibition assay	Inhibition of Epidermal Growth Factor Receptor (EGFR) tyrosine kinase	Homo sapiens	IC_{50} (nM)
69877	Biochemical inhibition assay	In vitro inhibition of Epidermal Growth Factor Receptor (EGFR) tyrosine kinase	Homo sapiens	IC_{50} (nM)

Table S2. Performance of the hybrid XGB model evaluated using scaffold-based (Bemis-Murcko) dataset splitting

XGB Hybrid (Scaffold Split)	
Metric	Value
Accuracy	76.40
Sensitivity	78.33
Specificity	74.43
AUC	0.84
MCC	0.53
Precision	0.76
Recall	0.78
F1-score	0.77
PR-AUC	0.84

Figure S1. Workflow illustrating the dataset curation and labelling process

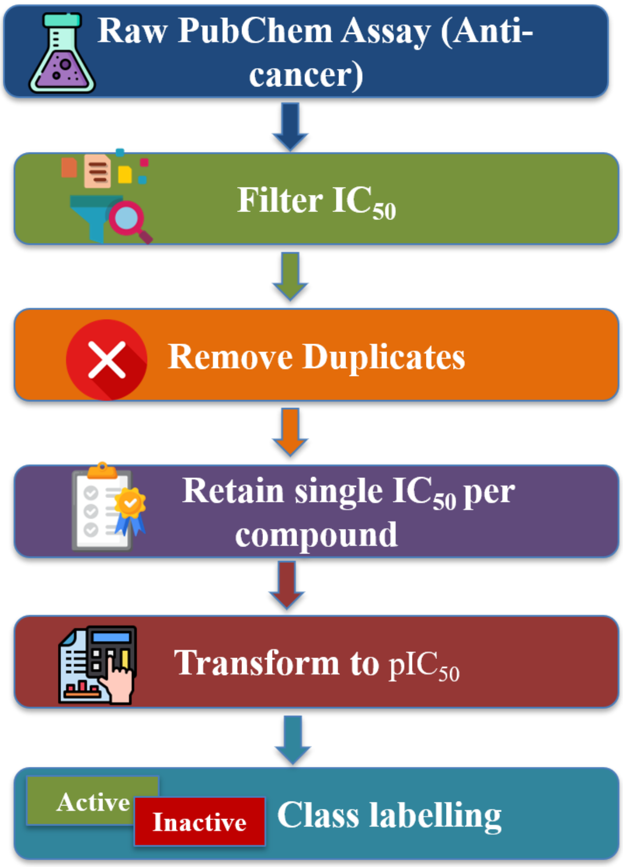

Supplement: Supplementary file 1 [file pharmaceuticals-19-00635-s001.zip › pharmaceuticals-4142267-supplementary.pdf]
